# Prevalence and Predictors of Diabetic Retinopathy in Saudi Arabia: Insights from a Systematic Review and Meta-Analysis

**DOI:** 10.3390/biom14121486

**Published:** 2024-11-22

**Authors:** Ali Mohammed Alshahrani, Alaa Mohammed Alshahrani, Beshayer Abdullah H. Al-Boqami, Alwaleed Abdulhadi Alqahtani, Bassam Alzahrani, Yousef Bassi, Mohammed Yousef Almohaimeed, Abeer Mohammed Alalmaai, Ariana Saraiva, Bandar Naffaa Alhumaidi, Najla A. Albaridi, Maria João Lima, Conrado Carrascosa, António Raposo

**Affiliations:** 1 Department of Ophthalmology, Armed Forces Hospital Southern Region, Khamis Mushit 62413, Saudi Arabia; dr.alishahrani@gmail.com; 2 Department of Family Medicine, Armed Forces Hospital Southern Region, Khamis Mushit 62413, Saudi Arabia; dr_alaa_m_sh@icloud.com; 3 Al Shifa Hospital, Qassim Health Cluster, Buraydah 52355, Saudi Arabia; dr.beshayer@gmail.com; 4 College of Medicine, Imam Mohammad Ibn Saud Islamic University, Riyadh 11623, Saudi Arabia; alwaleed8alqahtani@gmail.com; 5 College of Medicine, King Saud bin Abdulaziz University for Health Sciences (KSAU-HS), Riyadh 11481, Saudi Arabia; bassamzh.07@gmail.com; 6 College of Medicine, King Abdulaziz University, Jeddah 22252, Saudi Arabia; yousefbassi55@gmail.com; 7 College of Medicine, Qassim University, Buraydah 51452, Saudi Arabia; mohammed.yosef8899@gmail.com; 8 College of Medicine, King Khalid University, Abha 62529, Saudi Arabia; abeeeer2356@gmail.com; 9 Research in Veterinary Medicine (I-MVET), Faculty of Veterinary Medicine, Lisbon University Centre, Lusófona University, Campo Grande 376, 1749-024 Lisboa, Portugal; ariana.saraiva@ulusofona.pt; 10 Department of Community Health Nursing, College of Nursing, Taibah University, Al Madinah Al Munawwarah 42241, Saudi Arabia; bhumide@taibahu.edu.sa; 11 Department of Health Science, College of Health and Rehabilitation, Princess Nourah bint Abdulrahman University, P.O. Box 84428, Riyadh 11671, Saudi Arabia; naalbaridi@pnu.edu.sa; 12 CERNAS Research Centre, Polytechnic University of Viseu, 3504-510 Viseu, Portugal; mjoaolima@esav.ipv.pt; 13 Department of Animal Pathology and Production, Bromatology and Food Technology, Faculty of Veterinary, Universidad de Las Palmas de Gran Canaria, Trasmontaña s/n, 35413 Arucas, Spain; 14 CBIOS (Research Center for Biosciences and Health Technologies), Universidade Lusófona de Humanidades e Tecnologias, Campo Grande 376, 1749-024 Lisboa, Portugal

**Keywords:** diabetic retinopathy, glycemic control, meta-analysis, prevalence, public health interventions, risk factors, Saudi Arabia, systematic review, type 1 diabetes, type 2 diabetes

## Abstract

Background: Diabetic retinopathy (DR) is one of the leading causes of blindness among diabetic patients, particularly in areas with an increase in diabetes epidemics, such as Saudi Arabia. Notwithstanding the significant public health implications, data on the prevalence and risk factors of DR in Saudi Arabia are few and scattered, limited to certain geographic areas. Our study objective is to conduct a systematic review of the literature and a meta-analysis of the prevalence and predictors for DR in Saudi Arabia, within both type 1 and type 2 diabetes. Methods: A systematic review and meta-analysis were constructed according to PRISMA guidelines. We searched PubMed, Embase, Web of Science, and Google Scholar electronic databases for studies published from 2000–2023. Any study related to the prevalence of diabetic retinopathy in T1DM or T2DM among adult patients aged ≥18 years that was conducted in Saudi Arabia was included. Pooling prevalence estimates were calculated using a random-effects model, and heterogeneity across the studies was tested by the I^2^ statistic and Cochran’s Q test. Results: A total of 11 studies published between 2006 and 2019 met the inclusion criteria, with sample sizes ranging from 99 to over 50,000 participants. The overall pooled prevalence of DR was estimated to be 31% (95% CI: 24–39%), with substantial heterogeneity observed across studies (I^2^ = 99%). Prevalence estimates ranged from 16.7% to 69.8% and were influenced by variables such as study design, duration of diabetes, and glycemic control. Among individuals with type 2 diabetes, the pooled prevalence was 24% (95% CI: 20–28%). Poor glycemic control and longer diabetes duration were consistently identified as significant predictors of DR, while other factors, such as obesity and hypertension, were also associated with an increased risk of DR. Conclusions: The high prevalence of DR in Saudi Arabia highlights the critical need for focused public health initiatives, especially among those with type 2 diabetes. To minimize the effects of DR, early intervention, routine DR screening programs, and optimal diabetes control are essential. The increasing prevalence of DR in Saudi Arabia requires careful consideration of healthcare policy and resource allocation, which is made possible by our results.

## 1. Introduction

Chronic hyperglycemia is a hallmark of diabetes mellitus (DM), a long-term metabolic disease caused by deficiencies in either the production of insulin, the action of insulin, or both [1]. The International Diabetes Federation said that 463 million people worldwide suffered from diabetes in 2019. This indicates that the prevalence of the disease has increased to worrying proportions. By 2045, this figure is expected to increase to 700 million [2]. The Middle East and North Africa region has seen a particularly sharp increase in diabetes prevalence, driven by factors such as rapid urbanization, sedentary behaviors, and changes in dietary habits, contributing to the substantial rise in type 2 diabetes mellitus (T2DM) cases [3]. Particularly, the Kingdom of Saudi Arabia, which is ranked second in the Middle East and seventh internationally for diabetes prevalence, has a significant diabetes burden. Current statistics indicate that approximately seven million Saudi individuals are diagnosed with diabetes, with an additional three million in the prediabetes stage [4]. This high prevalence is attributed to various factors, including genetic predisposition, obesity, physical inactivity, and socioeconomic changes associated with rapid urbanization and westernization of lifestyles [5].

Diabetic retinopathy (DR), a microvascular consequence that continues to be the world’s primary cause of visual impairment and blindness in working-age people, is one of the most serious side effects of diabetes [6]. Chronic hyperglycemia damages the retinal blood vessels, resulting in increased vascular permeability, vessel blockage, and ischemia. This damage is the cause of diabetic retinopathy (DR) [7]. The progression of DR is gradual and often asymptomatic in its early stages, making regular screening essential for timely detection and intervention. Clinically, DR can manifest in various stages, ranging from mild non-proliferative changes characterized by heightened vascular permeability to more advanced stages. These include proliferative diabetic retinopathy (PDR), where aberrant blood vessel development occurs on the retina and the posterior surface of the vitreous humor, and severe non-proliferative diabetic retinopathy (NPDR), which is characterized by vascular closure [8]. Any stage of retinopathy can result in diabetic macular edema (DME), which is the swelling and possible loss of vision caused by fluid and lipid leakage from damaged blood vessels into the macula, the center section of the retina [9].

The global prevalence of DR among individuals with diabetes is estimated at 27.0%, representing a significant public health concern [10]. However, the prevalence can vary considerably across different regions and populations. In Saudi Arabia, previous studies have reported a wide range of DR prevalence, from 19.7% to 69.8%, among diabetic patients [11,12]. This variation emphasizes the necessity of a thorough and current evaluation of the incidence of DR in the kingdom. A number of risk variables, such as the length of diabetes, inadequate glycemic management, hypertension, dyslipidemia, nephropathy, and genetic factors, have been linked to the onset and progression of DR [13,14]. For the purpose of creating focused screening programs and therapies to lessen the burden of DR, it is essential to comprehend the prevalence and risk factors unique to the Saudi population.

There is a dearth of thorough national statistics on the prevalence of diabetic retinopathy (DR) and related risk factors, despite the disease’s substantial negative effects on public health and quality of life in Saudi Arabia. The majority of research has been conducted in localized regions or inside certain healthcare facilities, which has limited the applicability of its results to a larger community [15]. Additionally, rapid lifestyle changes and evolving healthcare practices in the kingdom underscore the need for an updated analysis of DR epidemiology. The heterogeneity in study methodologies, sample sizes, and diagnostic criteria further complicates the interpretation of existing data, making a systematic synthesis of available evidence crucial for a clearer understanding of DR prevalence in Saudi Arabia [16].

The complexity of DR management in Saudi Arabia is compounded by region-specific factors, including healthcare access challenges in rural areas, cultural beliefs that influence healthcare-seeking behaviors, and the genetic predisposition associated with consanguineous marriages [17]. Furthermore, the young demographic profile of the Saudi population, with a significant portion under the age of 30, raises concerns about the future burden of DR as this cohort ages and faces increased risks of diabetes-related complications [18]. The present study aims to give a complete assessment of the prevalence of DR among diabetic patients in Saudi Arabia, taking into account both type 1 and type 2 diabetes. The systematic review and meta-analysis will provide insights that may be used to guide future screening programs, public health policies, and targeted treatments.

## 2. Methods

This comprehensive systematic review and meta-analysis followed the guidelines set forth in the Preferred Reporting Items for Systematic Reviews and Meta-Analyses (PRISMA) [19], with all procedures carried out in accordance with the Cochrane Handbook [20].

### 2.1. Search Strategy and Selection Criteria

The systematic review and meta-analysis were conducted following the Preferred Reporting Items for Systematic Reviews and Meta-Analyses (PRISMA) guidelines. A thorough literature search was carried out across multiple electronic databases, including PubMed, Embase, Web of Science, and Google Scholar. The search strategy utilized a combination of Medical Subject Headings (MeSH) terms and free-text keywords related to diabetic retinopathy and Saudi Arabia. The search terms included various iterations of “diabetic retinopathy”, “diabetes complications”, “eye diseases”, and “Saudi Arabia”. To guarantee current and highly relevant data, the search was restricted to publications published between 1 January 2000 and 31 December 2023. To find any further research that could have been missed during the first search, the reference lists of the included studies and relevant review articles were also manually evaluated. To reduce the possibility of publishing bias, grey literature—such as government reports and conference abstracts—was also investigated.

### 2.2. Inclusion and Exclusion Criteria

Studies were included if they fulfilled the following criteria: (1) observational studies (cross-sectional, cohort, or case–control) that reported the prevalence of diabetic retinopathy; (2) studies conducted within Saudi Arabia; (3) studies involving adult participants (≥18 years) diagnosed with either type 1 or type 2 diabetes mellitus; (4) studies published in either English or Arabic; and (5) studies that employed standard diagnostic methods for diabetic retinopathy, such as fundus photography, fluorescein angiography, or ophthalmoscopy. We excluded studies if: (1) they were case reports, reviews, editorials, or conference abstracts; (2) focused solely on specific subpopulations (e.g., only gestational diabetes); (3) used unclear or non-standard diagnostic criteria for diabetic retinopathy; or (4) they were duplicate publications or secondary analyses of previously published data.

### 2.3. Study Selection

To guarantee that all pertinent studies on diabetic retinopathy (DR) in Saudi Arabia were thoroughly included, a rigorous research selection method was implemented for this systematic review and meta-analysis. Duplicate entries were eliminated after all recognized articles were loaded into EndNote for management. In order to exclude papers that were either irrelevant or did not fit the predetermined inclusion criteria, two independent reviewers went through the titles and abstracts. After passing the first screening, full-text articles were carefully assessed to determine their suitability. Any disputes amongst the reviewers were handled by conversation or, if required, contact with a third reviewer. The references of the chosen publications were thoroughly examined to make sure that no pertinent research was overlooked.

### 2.4. Data Collection

A systematic approach was utilized for data extraction, concentrating on the prevalence and characteristics of diabetic retinopathy among individuals with diabetes in Saudi Arabia. Key variables extracted from each study included the study design, sample size, type of diabetes (type 1 or type 2), diagnostic methods used for identifying diabetic retinopathy, and reported prevalence rates. Additional data points included patient demographics, duration of diabetes, glycemic control, and any identified risk factors for DR. Each study was carefully evaluated to ensure sufficient data were available for inclusion in the meta-analysis.

### 2.5. Quality Assessment

An established tool for evaluating the quality of observational studies, the Newcastle–Ottawa Scale (NOS), was used to assess the quality of the included studies [21]. Every study was assessed by two independent and unbiased reviewers in three primary areas: choosing study groups, group comparability, and result evaluation. The quality of the studies was classified as high, moderate, or poor based on how well they met the NOS criteria. All disagreements among the reviewers were settled by dialogue, guaranteeing precision and uniformity in the process of evaluating the quality of all the included studies.

### 2.6. Statistical Analysis

For this systematic review and meta-analysis, MetaXL version 5.3 (EpiGear International, Sunrise Beach, Australia) was used for the statistical analysis [22]. A random-effects model was employed using the inverse variance method to calculate the pooled prevalence of diabetic retinopathy (DR) among diabetes patients in Saudi Arabia. This model was selected to account for the variability anticipated among the included studies. The pooled prevalence was reported with 95% confidence intervals (CIs). Heterogeneity among the studies was assessed using the Higgins I^2^ statistic, with values above 50% indicating substantial heterogeneity. Additionally, Cochran’s Q test was performed to further evaluate heterogeneity. The results were visually represented using forest plots, displaying individual and pooled prevalence rates with their corresponding confidence intervals. MetaXL provided a reliable framework for analyzing the meta-analytic data and summarizing the study outcomes.

## 3. Results

### 3.1. Article Screening and Selection

**Figure 1:**
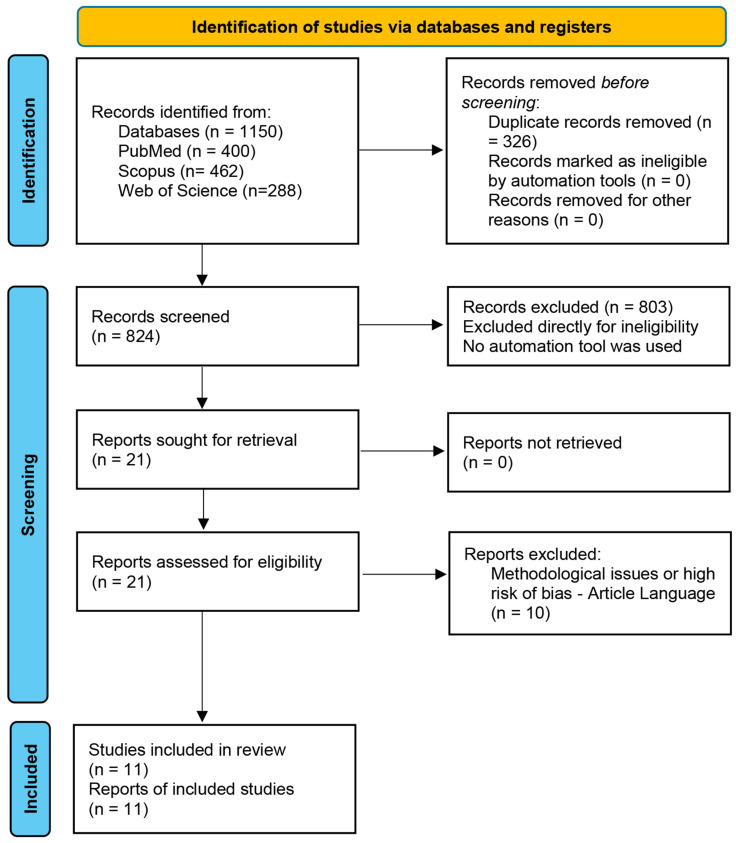
PRISMA flow diagram. PRISMA = Preferred Reporting Items for Systematic Reviews and Meta-Analyses.

A thorough literature search yielded 1150 records from various databases and other sources. After the removal of duplicates, 824 unique records remained for screening. During this phase, 791 records were excluded for not meeting the relevance criteria, with 770 studies deemed off-topic and 33 classified as non-research articles. After this screening process, 21 studies were selected for full-text review to assess their eligibility for inclusion. Following the full-text review, 10 studies were excluded due to methodological limitations, high risk of bias, or failure to meet language requirements. Ultimately, 11 studies satisfied the predefined inclusion criteria and were incorporated into the systematic review and meta-analysis [12,23,24,25,26,27,28,29,30,31,32] (see Figure 1).

### 3.2. Characteristics of the Included Studies

The 11 studies included in this meta-analysis were conducted between 2006 and 2019 across various clinical settings in Saudi Arabia, with sample sizes ranging from 99 to over 50,000 participants. While most studies focused primarily on patients with type 2 diabetes, several also included individuals with type 1 diabetes. The median age of participants generally ranged from their 50s to 60s, and the duration of diabetes varied across studies, with some reporting an average disease duration of up to 13 years. The prevalence of diabetic retinopathy (DR) showed significant variation across the studies, ranging from 16.7% in the study by Alwakeel et al. (2008) [32] to 69.8% in the study by Al-Otaibi et al. (2017) [30]. Several studies highlighted the role of poor diabetes control, measured by HbA1c levels, as a key factor in DR prevalence. For example, Ahmed et al. (2016) [29] reported that 42.4% of participants had poor control, correlating with a DR prevalence of 36.4%. Non-proliferative diabetic retinopathy (NPDR) was more common than proliferative diabetic retinopathy (PDR), as seen in Khan et al. (2010) [12], where 27.7% had NPDR and only 2.3% had PDR. The duration of diabetes was consistently linked to higher DR prevalence, emphasizing the need for early detection and intervention. Additionally, many studies reported that the majority of participants were overweight or obese, with a mean BMI exceeding 30 kg/m^2^, highlighting the importance of addressing obesity in managing diabetes and its complications. Table 1 provides detailed characteristics of the 11 studies.

### 3.3. Pooled Estimates of Diabetic Retinopathy Among the General Diabetic Population

**Figure 2:**
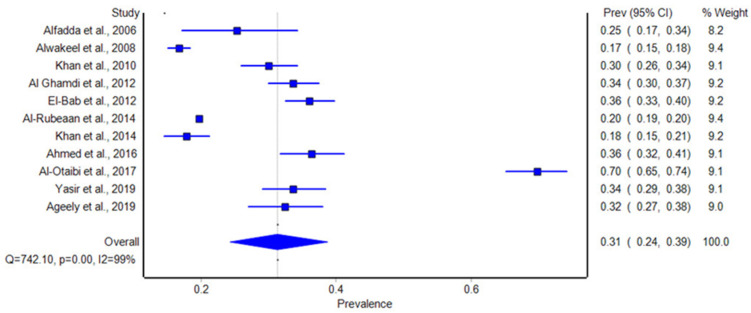
Forest plot of the prevalence of diabetic retinopathy among patients with diabetes of our included studies. Blue square boxes represent the rate [12,23,24,25,26,27,28,29,30,31,32].

The meta-analysis showed that Saudi Arabia’s general diabetic population had a high overall prevalence of diabetic retinopathy (see Figure 2). The pooled estimate of DR prevalence across the 11 included studies was 31%, with a 95% confidence range (CI) of 24% to 39%. The prevalence, however, differed greatly between the studies; Alwakeel et al. (2008) [32] reported the lowest rate, at 16.7%, and Al-Otaibi et al. (2017) [30] reported the greatest rate, at 69.8%.

### 3.4. Pooled Estimates of Diabetic Retinopathy Among Type 2 Diabetes Patients

**Figure 3:**
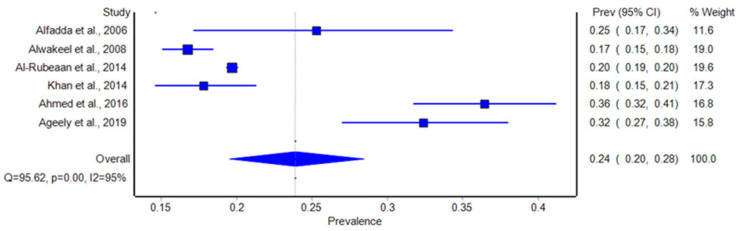
Forest plot of the prevalence of diabetic retinopathy among patients with type 2 diabetes in our included studies. Blue square boxes represent the rate [25,26,27,28,29,32].

Focusing specifically on patients with type 2 diabetes, the meta-analysis revealed a slightly lower pooled prevalence of diabetic retinopathy compared to the overall diabetic population. Among the studies that exclusively assessed type 2 diabetes patients, the pooled prevalence of DR was 24%, with a 95% confidence interval of 20% to 28%. The range of reported prevalence in this subgroup also varied, with Alwakeel et al. (2008) [32] documenting the lowest prevalence at 16.7% and Ageely et al. (2019) [28] reporting a higher prevalence of 32.4%. Similar to the overall population, significant heterogeneity was observed in this subgroup (I^2^ = 95%), indicating that study-specific factors, including differences in population demographics, duration of diabetes, and healthcare access, contributed to the variability in DR prevalence. Despite this variation, the findings suggest that nearly a quarter of type 2 diabetes patients in Saudi Arabia are affected by DR (as shown in Figure 3).

### 3.5. Subgroup Analysis Across Healthcare Settings

**Figure 4:**
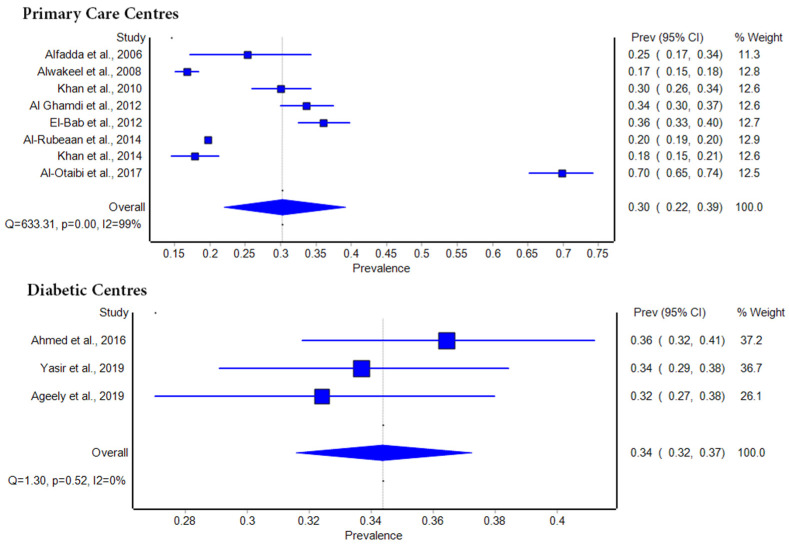
Subgroup analysis of diabetic retinopathy prevalence across primary care [12,23,25,26,27,30,31,32] and diabetic centers in Saudi Arabia [24,28,29].

Figure 4 presents a subgroup analysis of diabetic retinopathy (DR) prevalence across different healthcare settings, specifically primary care centers and diabetic centers. This analysis highlights the variability in DR prevalence based on the type of healthcare facility where patients were managed. In primary care centers, the pooled prevalence of DR was estimated at 30% (95% CI: 22–39%), with individual studies showing a wide range of prevalence estimates from 17% to 70%. Studies by Alfadda et al. (2006) and Al-Otaibi et al. (2017) reported the lowest and highest prevalence, respectively [27,30]. The significant heterogeneity in this subgroup (I^2^ = 99%) suggests considerable variability in patient characteristics and healthcare practices across primary care settings. In contrast, studies conducted in diabetic centers yielded a pooled prevalence of 34% (95% CI: 32–37%), with less variability between studies. Prevalence estimates ranged from 32% to 36%, with individual studies by Ahmed et al. (2016), Yasir et al. (2019), and Ageely et al. (2019) contributing to this analysis [24,28,29]. The lower heterogeneity in this subgroup (I^2^ = 0%) suggests more consistent patient management and DR screening protocols within specialized diabetic centers compared to primary care settings.

### 3.6. Study Bias and Sensitivity Identification

**Figure 5:**
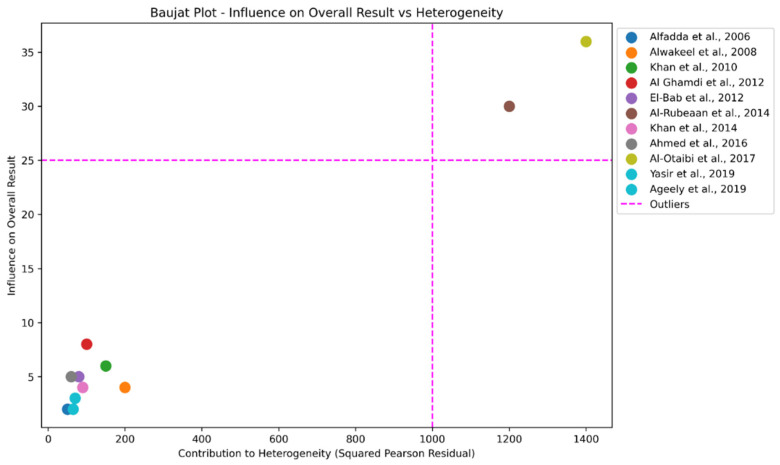
Baujat plot highlighting study contributions to heterogeneity and influence on overall diabetic retinopathy prevalence estimates [12,23,24,25,26,27,28,29,30,31,32].

The Baujat plot (Figure 5) highlighted two studies, Al-Rubeaan et al., 2014 and Al-Otaibi et al., 2017, as significant outliers contributing disproportionately to heterogeneity and strongly influencing the overall result. Specifically, Al-Rubeaan et al., 2014 reported a prevalence of 19.7% with a confidence interval (CI) of 0.19 to 0.20 among a large sample of 50,464 participants, while Al-Otaibi et al., 2017 showed a markedly higher prevalence of 69.8% (CI: 0.65 to 0.74) in a much smaller cohort of 400 participants [26,30].

The placement of these studies in the upper-right quadrant of the Baujat plot indicates their substantial contribution to both heterogeneity and influence, suggesting potential sources of bias in the pooled estimate. To assess the impact of these outliers, we performed a sensitivity analysis by excluding Al-Rubeaan et al., 2014 and Al-Otaibi et al., 2017 from the meta-analysis [26,30]. Initially, the pooled prevalence of diabetic retinopathy (DR) was 31% (CI: 0.24 to 0.39), with high heterogeneity (I^2^ = 99%, Q = 742.10, *p* < 0.001).

After excluding these studies, the revised pooled prevalence was 28.7% (CI: 0.22 to 0.36), with a reduced heterogeneity, as indicated by an I^2^ of 96.2% and a Cochran’s Q of 212.41. The reduction in heterogeneity demonstrates that these outliers significantly contributed to the initial variability in the results.

### 3.7. Study-Level Quality Assessment

The quality of the included studies was evaluated using a standardized scoring system, which assessed the selection methods, comparability of the study groups, and the accuracy of exposure or outcome assessments. Each study was rated based on these criteria to ensure a consistent and rigorous evaluation of study quality (see Table 2). This approach allowed for the identification of potential biases and methodological limitations across the included studies. Of the eleven studies included in the meta-analysis, six were rated as high quality, achieving scores between 7 and 9 points. Alwakeel et al. (2008) and Al-Rubeaan et al. (2014) achieved the highest scores, indicating optimal methodological rigor, particularly in their selection methods and outcome assessment [26,32]. The remaining five studies were categorized as moderate quality, with scores ranging from 6 to 7 points. These studies typically lost points due to issues related to study design, such as the absence of detailed comparability between study groups or limitations in outcome assessment. The variability in study quality reflects differences in the rigor of the research methodologies employed, with some studies lacking in comparability or detail in the assessment of diabetic retinopathy.

## 4. Discussion

The systematic review and meta-analysis provide an important contribution to our understanding of the prevalence and risk factors for diabetic retinopathy (DR) in Saudi diabetics. With data from 11 studies over a 13-year period, it is one of the most thorough evaluations to date. The results demonstrate the significant prevalence of diabetic retinopathy (DR) in Saudi Arabia, especially in individuals with poor glycemic control, long-standing diabetes, and other concomitant diseases such as obesity and hypertension.

The pooled prevalence of diabetic retinopathy (DR) across the included studies was 31%, though there was considerable variation, with prevalence rates ranging from 16.7% to 69.8%. This variability can largely be explained by differences in study design, sample sizes, population characteristics, and the types of healthcare settings where the studies were conducted. For example, studies carried out in specialized diabetes care centers, such as the study by Al-Otaibi et al. (2017), tended to report higher prevalence rates compared to studies conducted in primary healthcare settings, like the study by Alwakeel et al. (2008) [30,32]. Internationally, DR prevalence also shows wide regional differences. In South Asia, the prevalence ranges from 18% in Chennai to 38.8% in Nepal and as high as 60.9% in certain Indian populations [33,34,35,36]. In developed countries, such as the United Kingdom and the United States, DR prevalence is reported at 39.5% and 42%, respectively, particularly among minority groups [37,38]. In Africa, studies show prevalence rates around 37%, while in the eastern Mediterranean region, the prevalence reaches 31%, slightly higher than the global average of 25.2% [39,40]. These variations underscore the impact of healthcare access, socioeconomic factors, and regional healthcare infrastructure on the prevalence of diabetic retinopathy. Additionally, they highlight the critical need for standardized screening protocols and diagnostic criteria for DR, as well as enhanced access to care and early detection programs [40]. Tailored public health interventions are essential to address these disparities and improve outcomes for diabetic patients across different regions.

This study found that poor glycemic control, which is typically assessed by HbA1c levels, was one of the major predictors of diabetic retinopathy (DR). Ahmed et al. (2016), for example, revealed that 42.4% of individuals with poor glycemic control developed DR. This conclusion is consistent with global studies showing that raised HbA1c levels are closely connected with complications related to diabetes, such as retinopathy [29]. Similarly, a systematic review and meta-analysis conducted by Narsaiah et al. (2019) further confirmed that poor glycemic management greatly increases the risk of DR by reiterating the strong link between higher HbA1c levels and DR prevalence [41,42]. Strong evidence that rigorous glycemic control can dramatically lower the risk of diabetic kidney disease in individuals with type 1 diabetes was also provided by the Diabetes Control and Complications Trial (DCCT). This trial has shown that the chance of developing DR dropped by 35% for every 1% reduction in HbA1c [36]. These data demonstrate the need for comprehensive blood glucose management to help reduce the risk of diabetic retinopathy and underline the critical role that stringent glycemic control plays in delaying the start and progression of DR, particularly in those with long-standing diabetes.

Another significant risk factor identified was the duration of diabetes. Prolonged exposure to hyperglycemia is known to cause both macrovascular and microvascular damage, leading to complications such as retinopathy, nephropathy, and neuropathy. Most studies included in this review indicated that a longer duration of diabetes was associated with a higher risk of DR. For example, studies reporting on populations with a longer duration of diabetes, such as Al-Rubeaan et al. (2014), tended to show higher DR prevalence rates. This finding is consistent with global data, which indicate that each additional year of diabetes increases the risk of developing diabetic retinopathy (DR) by approximately 6% [26]. These results highlighted how crucial it is to manage diabetes early and well in order to reduce the risk of long-term consequences, such as the onset and progression of DR. Patients with diabetes can see a marked reduction in the burden of this condition when early and effective treatment techniques are used.

Apart from diabetes duration and glycemic control, obesity and hypertension were found to be important risk factors for diabetic retinopathy (DR). The populations under study had a very high prevalence of obesity, with mean body mass indices (BMIs) often surpassing 30 kg/m^2^. It is commonly known that obesity increases the incidence of type 2 diabetes and all of its aftereffects, including DR. Obesity was substantially linked to a greater frequency of DR, according to a meta-analysis evaluating the association between DR risk and obesity. The relative risk (RR) was 1.20 (95% CI, 1.01–1.43: I^2^ = 59.6%) [43]. Furthermore, studies like those by Alwakeel et al. (2008) and Al Ghamdi et al. (2012) showed a substantial association between increased blood pressure and DR prevalence, supporting the notion that hypertension is significantly associated with the development of DR [31,32]. In order to avoid diabetic retinopathy (DR) and to lessen the more serious problems linked to diabetes, it is imperative that people with diabetes have their cardiovascular risk factors addressed.

The high level of heterogeneity observed in this meta-analysis (I^2^ = 99%) reflects substantial variation in study populations, clinical settings, and methodologies. While such variability is a common challenge in systematic reviews and meta-analyses, it suggests that the true burden of diabetic retinopathy (DR) may differ significantly across various regions and populations within Saudi Arabia. Factors such as differences in healthcare access, the availability of screening programs, and disparities in clinical practices likely contribute to these discrepancies. To address these variations and improve patient outcomes, it is essential to implement standardized diagnostic criteria and uniform screening protocols across the country. This would help ensure that all individuals with diabetes receive timely, accurate diagnoses and appropriate treatment for DR.

This study’s findings also have important public health implications. The rising prevalence of diabetes in Saudi Arabia, driven by increasing rates of obesity and sedentary lifestyles, suggests that the burden of DR will continue to grow in the coming years. Policymakers and healthcare providers must prioritize the prevention and early detection of DR through public health campaigns, patient education, and improved access to eye care services. The importance of primary care doctors in the early detection and treatment of diabetes and its complications should be highlighted in particular. Routine treatment procedures should include regular eye exams for diabetic patients, particularly those with poor glucose control or long-term diabetes.

### 4.1. Recommendations

For future primary studies on diabetic retinopathy (DR) in Saudi Arabia, we recommend expanding data collection across diverse regions, especially rural and underserved areas, to improve the representativeness and generalizability of findings. Current research primarily targets urban populations, which may not capture differences in healthcare access, diabetes prevalence, or DR risk factors found in other regions. Including data from these areas would provide a fuller picture of DR prevalence and help inform targeted healthcare policies. Future studies should also conduct subgroup analyses based on key factors such as age, diabetes duration, and glycemic control levels to provide specific insights into high-risk populations. Such stratification is essential for designing effective screening and intervention programs tailored to distinct demographic groups. Importantly, we encourage healthcare authorities to establish and adopt standardized diagnostic criteria and methodologies across all healthcare settings. This consistency would improve the comparability of study findings and support more reliable pooled estimates in future meta-analyses. Standardized diagnostic approaches would enable future research efforts to produce results that are directly comparable, thereby enhancing the utility of meta-analytic reviews and informing more precise healthcare strategies.

Given the distinct pathogenesis and disease progression in type 1 and type 2 diabetes, future studies should analyze DR risk factors separately in these populations. This separation would provide a clearer understanding of risk profiles unique to each diabetes type and support the development of targeted prevention and treatment strategies. Moreover, recognizing the strong link between glycemic control and DR progression, we recommend that future research consistently include data on glycemic management practices, such as HbA1c levels, and incorporate these as central variables in study designs. Investigating the impact of patient education, self-management adherence, and lifestyle factors (e.g., diet, exercise) would provide actionable insights into DR prevention strategies. Encouraging a standardized approach and adherence to best practices across studies will establish a robust, evidence-based foundation for improved DR screening, prevention, and management across diverse populations within Saudi Arabia’s healthcare system.

### 4.2. Limitations

Although this study provides valuable insights, several limitations should be acknowledged. The included studies exhibited a high degree of heterogeneity, with considerable variation in sample sizes, study designs, and diagnostic criteria. Most of the studies were cross-sectional, which restricts the ability to draw causal conclusions. Additionally, since the majority of studies were conducted within Saudi Arabia, the findings may not be fully generalizable to other regions within the Gulf Cooperation Council (GCC) countries. Future research should aim to incorporate a wider range of studies from different countries in the region and adopt more standardized methodologies to enhance the comparability of results.

## 5. Conclusions

The substantial burden of diabetic retinopathy (DR), with an overall prevalence of 31% in Saudi Arabia, is highlighted by this systematic review and meta-analysis. DR is more common in those with long-standing diabetes, poor glycemic control, and concomitant conditions such as obesity and hypertension. These results highlight how crucial it is to increase screening programs, encourage early identification, and enhance diabetes care to lower the prevalence of DR. In order to minimize complications from diabetic retinal disease (DR) and to improve outcomes for the increasing number of diabetics in Saudi Arabia, it is imperative that modifiable risk factors be addressed and that diabetic patients have frequent eye examinations.

## Figures and Tables

**Table 1:** Characteristics of the studies about diabetic retinopathy among patients with diabetes in Saudi Arabia.

Study Reference	Year	Population	Study Design	Sample Size	Diabetes Type	DR Cases	DR Prevalence (%)	NPDR Cases (%)	PDR Cases (%)	Median Age (Years)	Age Variation (Years)	Duration of Diabetes (Years)	Diabetes Control	BMI
Alfadda et al. [27]	2006	Patients attending primary care clinics at KKUH over a 3-year period	Retrospective chart review	99	Type 2	25	25.3	NA	NA	57.0	NA	11.8	24.7% HbA1c ≤ 7.0	Mean: 30.8 kg/m^2^
Alwakeel et al. [32]	2008	Type 2 diabetes patients at Security Forces Hospital, Riyadh	Retrospective review of medical records	1952	Type 2	326	16.7	11.4	5.3	58.4	14.2	10.4 ± 7.5	NA	Mean: 29.89 kg/m^2^
Khan et al. [12]	2010	Patients from multiple clinics	Cross-sectional study	473	Types 1 and 2	142	30.0	27.7	2.3	NA	NA	8.6 ± 6.0	36.3% uncontrolled	Mean: 30.63 kg/m^2^
Al Ghamdi et al. [31]	2012	Patients aged 50+ in Taif, Saudi Arabia	Cross-sectional study	612	NA	206	33.7	31.0	3.5	63.3	NA	NA	NA	NA
El-Bab et al. [23]	2012	Patients with diabetes from multiple hospitals	Cross-sectional study	690	Types 1 and 2	249	36.1	29.7	6.4	46.1	11.9	11.9 ± 7.9	NA	NA
Al-Rubeaan et al. [26]	2014	Patients from the Saudi National Diabetes Registry	Cross-sectional study	50,464	Type 2	9936	19.7	9.1	10.6	59.7	12.8	13.4 ± 8.2	NA	NA
Khan et al. [25]	2014	Patients from primary health centers in the Al Hasa region	Cross-sectional study	506	Type 2	90	17.8	18.3	1.4	57.4	NA	10.2 ± 6.0	NA	Mean: 30.11 kg/m^2^
Ahmed et al. [29]	2016	Type 2 diabetes patients attending Abha Diabetic Center	Cross-sectional study	401	Type 2	146	36.4	32.2	4.2	54.6	12.3	13.4 ± 7.9	Poor control: 42.4%	Mean: 31.14 kg/m^2^
Al-Otaibi et al. [30]	2017	Patients attending primary care clinics	Retrospective study	400	Types 1 and 2	279	69.8	NA	NA	NA	NA	NA	NA	NA
Yasir et al. [24]	2019	Patients attending diabetes clinics in Riyadh	Cross-sectional study	395	Types 1 and 2	133	33.7	39.7	5.0	NA	NA	NA	NA	NA
Ageely et al. [28]	2019	Patients attending Jazan Diabetes Centre	Cross-sectional study	281	Type 2	91	32.4	NA	NA	NA	NA	NA	Mean HbA1c: 9.24	Mean: 28.79 kg/m^2^

NA: Not available.

**Table 2:** Quality assessment of the included studies.

Study (Author, Year)	Selection (Max 4 Points)	Comparability (Max 2 Points)	Exposure/Outcome (Max 3 Points)	Total Score (Max 9 Points)	Quality Tier
Alfadda et al., 2006 [27]	3	1	2	6	Moderate
Alwakeel et al., 2008 [32]	4	2	3	9	High
Khan et al., 2010 [12]	3	1	2	6	Moderate
Al Ghamdi et al., 2012 [31]	4	1	2	7	High
El-Bab et al., 2012 [23]	3	1	2	6	Moderate
Al-Rubeaan et al., 2014 [26]	4	2	3	9	High
Khan et al., 2014 [25]	3	1	2	6	Moderate
Ahmed et al., 2016 [29]	4	2	3	9	High
Al-Otaibi et al., 2017 [30]	3	1	2	6	Moderate
Yasir et al., 2019 [24]	4	2	3	9	High
Ageely et al., 2019 [28]	4	2	3	9	High

## Data Availability

Not applicable.
